# Methionine Dependency and Restriction in Cancer: Exploring the Pathogenic Function and Therapeutic Potential

**DOI:** 10.3390/ph18050640

**Published:** 2025-04-28

**Authors:** Chi Ma, Aoshuang Xu, Liping Zuo, Qun Li, Fengjuan Fan, Yu Hu, Chunyan Sun

**Affiliations:** Department of Hematology, Union Hospital, Tongji Medical College, Huazhong University of Science and Technology, Wuhan 430022, China; machi262300@163.com (C.M.); xas_medicine@163.com (A.X.); zuo5258@126.com (L.Z.); liqun97@126.com (Q.L.); fengjuan_fan@hust.edu.cn (F.F.); dr_huyu@126.com (Y.H.)

**Keywords:** methionine metabolism, cancer, epigenetic modification, tumor microenvironment, methionine restriction

## Abstract

Methionine, an essential amino acid, is obtained by dietary intake to fulfill the requirements of our bodies. Accumulating evidence indicates that methionine plays a pivotal role in various biological processes, including protein synthesis, energy metabolism, redox balance maintenance, and methylation modifications. Numerous advances underscore the heightened dependence of cancer cells on methionine, which is a significant factor in cancer pathogenesis and development. A profound comprehension of the intricate relationship between methionine metabolism and tumorigenesis is imperative for advancing the field of cancer therapeutics. Herein, we delve into the role of methionine in supporting cancer growth, the impact on epigenetic modifications, and the interaction between methionine and the tumor microenvironment. Additionally, we provide insights into the development of various methionine-targeted therapy strategies. This paper summarizes the current state of research and its translational potential, emphasizing the challenges and opportunities associated with harnessing methionine dependence as a target for innovative cancer treatments.

## 1. Introduction

Methionine, an essential sulfur-containing amino acid in mammals, participates in cell differentiation, tissue development, organ function, and the progression of diseases such as obesity and cancer. The primary metabolite of methionine, S-adenosyl-methionine (SAM), is involved in the methylation process of DNA, RNA, lipids, histones, and other proteins. Previous studies have revealed that most cancer cells exhibit an increased demand for methionine, which represents a common metabolic reprogramming phenomenon termed the Hoffmann effect [1,2]. Cancer cells cannot survive without methionine because of the arrested cell cycle and increased apoptosis, even when supplemented with homocysteine (Hcy) [3,4,5]. Conversely, normal cells can effectively utilize homocysteine to sustain growth without methionine [6].

Anomalous cancer metabolism is a recurring phenomenon throughout the progression of cancer. Recent studies have demonstrated that the accumulation of abnormal metabolites can facilitate the early development of cancers. Simultaneously, oncogenic alterations in the tumor microenvironment (TME) induce metabolic reprogramming, modifying multiple metabolic pathways to satisfy the immense bioenergetic and biosynthetic consumption in malignant cells [7,8]. Thus, the altered extracellular methionine uptake may present tumorigenesis and progression [9,10]. These preceding points indicate a promising opportunity in cancer to explore the vulnerability demonstrated by methionine. This paper will focus on the role of methionine and its metabolism during the development of cancer, encompassing the current understanding of how methionine contributes to the disease. Finally, we will provide a comprehensive summary of potential strategies targeting methionine metabolism, aiming to facilitate the development and clinical application of targeted drugs in the future.

## 2. Methionine Metabolism

### 2.1. Methionine Metabolic Pathway

**Figure 1:**
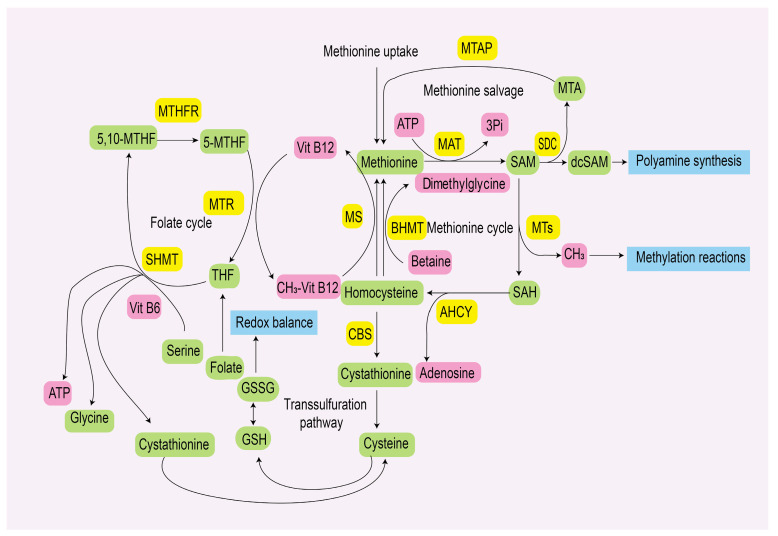
Methionine metabolism pathway. Methionine adenosyltransferases (MATs) catalyze the conversion of methionine and ATP into S-adenosylmethionine (SAM). Methyltransferases (MTs) mediate SAM transmethylation, generating S-adenosylhomocysteine (SAH). Alternatively, SAM undergoes decarboxylation via SAM decarboxylase (SAMDC), yielding decarboxylated SAM (dcSAM). Adenosylhomocysteinase (AHCY) hydrolyzes SAH, producing homocysteine and adenosine. Cystathionine-β-synthase (CBS) converts homocysteine into cystathionine in the transsulfuration pathway; subsequent cleavage yields cysteine. Homocysteine is remethylated to methionine by methionine synthase (MS), completing the methionine cycle. Alternatively, betaine–homocysteine methyltransferase (BHMT) utilizes betaine to remethylate homocysteine, generating methionine. In the methionine salvage pathway, methylthioadenosine phosphorylase (MTAP) reconverts 5-methylthioadenosine (MTA) into methionine. Within the folate cycle, methyl group transfer occurs sequentially between three key folate derivatives: tetrahydrofolate (THF), 5,10-methylenetetrahydrofolate (5,10-MTHF), and 5-methyltetrahydrofolate (5-MTHF). The 5-MTHF-derived methyl group remethylates homocysteine, thereby closing the methionine cycle. Serine hydroxymethyltransferase (SHMT) irreversibly cleaves serine into glycine and 5,10-MTHF.

Methionine becomes available in the metabolic pathway following the consumption, digestion, and absorption of food. According to current understanding, methionine undergoes catabolism and synthesis through multiple interconnected and tightly regulated mechanisms. In the methionine cycle, methionine adenosyltransferases (MATs) catalyze the synthesis of S-adenosylmethionine (SAM), the primary cellular methyl donor, which participates in methylation processes essential for cellular growth and function [11]. Moreover, SAM can also be converted into decarboxylated SAM (dcSAM), which acts as a donor of aminopropyl groups in polyamine biosynthesis, sustaining cancer cell proliferation and malignant transformation. Homocysteine, a key methionine metabolite, contributes to glutathione synthesis and maintains redox homeostasis via the transsulfuration pathway. Homocysteine, polyamines, and the production of SAM synthesis, 5-methylthioadenosine (MTA) contribute to endogenous methionine synthesis. The methionine cycle is inseparable from one-carbon metabolism, establishing direct connections among serine, glycine, and folate to maintain stable metabolite concentrations [12] (Figure 1).

A notable link exists between methionine and other essential amino acids. Fang et al. [13] demonstrated that mitochondria transmit methionine-related signals to maintain the balance between energy production and anabolism, which regulates cell proliferation. Lipoate metabolism is disrupted when SAM levels are low, leading to the rerouting of glucose-derived metabolites, such as acetyl-CoA, towards synthesizing other amino acids, especially arginine and leucine.

MAT I and MAT III, encoded by MAT1A, are predominantly expressed in the liver, leading to an increased synthesis of SAM. Conversely, MAT II, encoded by MAT2A, is expressed in most other cell types, where it plays a crucial role in the methionine cycle. Interestingly, methionine levels and MAT2A expression exhibit complex relationships across different tissues and cell types, which cannot be easily generalized. Some studies suggest that high expression of MAT2A occurs in response to the sharp decrease in SAM levels caused by methionine deficiency [14]. Other studies have found that at low methionine concentrations, MAT2B activates MAT2A by increasing the affinity of MAT2A to methionine. In contrast, when methionine or SAM levels are elevated, MAT2B inhibits MAT2A by reducing its enzymatic activity and increasing sensitivity to product inhibition [15].

SAMTOR, recently identified as a SAM sensor, connects methionine metabolism with the mechanistic target of rapamycin complex 1 (mTORC1) signaling pathway. As a consequence of methionine deficiency, reduced SAM levels facilitate the interaction between SAMTOR and GATOR1 (the GTPase activating protein for RagA/B), inhibiting mTORC1 signaling. Another study has shown that the mTORC1-c-Myc-SIRT4 axis modulates MAT2A activity through regulating the ADP-ribosylation of a glutamic residue [16]. By fostering endogenous methionine synthesis and decreasing methionine metabolism, this mechanism allows the methionine cycle to remain in balance [14,16,17,18,19,20]. Apart from SAMTOR, a recent study has identified an mTOR-independent regulatory mechanism that enables cells to survive under SAM depletion conditions and prevent excessive proliferation when SAM is abundant [21]. Unfortunately, the precise details of how this mechanism functions remain elusive.

### 2.2. Methionine Uptake Pathway

Solute carrier (SLC) transporters are responsible for the transport of nutrients and metabolites in the cells, including amino acids, vitamins, ions, and other essential molecules, and participate in metabolic reprogramming [22]. SLC transporters have more than 450 membrane-binding proteins with varying affinities for different amino acids. Multiple transporters in cells can simultaneously facilitate the transport of specific amino acids, while a single transporter may be expressed on different cell surfaces and cellular organelles. It has been found that the transporters solute carrier family 43 member 2 (SLC43A2), solute carrier family 7 member 5 (SLC7A5), and solute carrier family 3 member 2 (SLC3A2) play crucial roles in the methionine uptake pathway. NF-κB silencing results in a reduction in the expression of the methionine transporter SLC43A2, thereby downregulating methionine uptake in esophageal squamous cell carcinoma (ESCC) [23]. SLC7A5 (CD98 light chain) and SLC3A2 (CD98 heavy chain), components of L-type amino acid transporters, form the LAT1 heterodimer, which facilitates methionine transport [24]. In response to antigen stimulation, T cells upregulate the expression of SLC7A5, thereby increasing methionine uptake and promoting T cell activation [25]. However, the transport mechanisms and substrate affinities of most amino acid transporters remain poorly understood.

## 3. Methionine Metabolism in Cancer Cells

Excessive uptake of exogenous methionine is observed in various cancer types, including ESCC, glial tumors, multiple myeloma, and others [26,27]. A recent cohort study has identified evidence of four significant differential metabolites (namely pyruvate, glutamine, methionine, and lysine) in normal human oral epithelial cells (HOEC) and dysplastic oral keratinocyte (DOK) cells. A significant increase in intracellular methionine concentration has been observed following the transition from HOEC to DOK, suggesting that methionine demand rises with the progression of cell malignancy [9]. Dating back to 1983, researchers noted that ^11^C-methionine positron emission tomography (PET) is significantly more effective than ^68^Ga-EDTA and ^11^C-glucose PET in detecting brain cancer lesions due to the heightened methionine uptake by cancer cells [28]. Subsequently, numerous studies have shown that ^11^C-methionine PET exhibits a pronounced capacity for detecting various cancer foci, including lung cancer, breast cancer, and multiple myeloma, with a high detection rate and potential clinical relevance [26,29,30,31].

Accumulating evidence reveals that the endogenous methionine synthesized by methionine-dependent malignant cells is insufficient to meet the metabolic demands due to a possible lack of methionine synthase [32,33]. This phenomenon is further evidenced by elevated transmethylation activity and increased demand for SAM in cancer cells [32,34,35,36]. The methionine-dependent malignant cells in a methionine-restricted environment display a diminished SAM-to-SAH ratio, reduced transmethylation rate, and decreased overall cell methylation levels, notably in DNA methylation. However, the aforementioned effects can be reversed by supplying exogenous methionine [2,37,38,39]. As research progressed, methionine-restricted culture conditions resulted in significantly reduced histone methylation levels in cancer cells, while having only minimal effects on normal cells. It suggests that methionine addiction in cancer cells may also be associated with the instability of histone methylation [40].

James et al. [5] found that the growth and survival of all cancer cells are heavily dependent on exogenous methionine, although the degree of dependence varies among different cancer cell types. They initially tested 23 malignant cell lines in vitro and revealed that most experienced growth arrest, with only three demonstrating minimal proliferation. Subsequent studies have primarily focused on this cancer-specific methionine dependence, further validating the concept. Nevertheless, the molecular mechanisms underlying the differential methionine dependence observed across various cancer types remain elusive.

According to the findings, methionine impacts the cell cycle of methionine-dependent malignant cells. Methionine depletion results in an increased proportion of cancer cells in the G1 phase and a decrease in the S and G2-M phases [41,42]. The primary mechanism underlying G1 phase arrest involves the reduction in SAM induced by methionine deficiency. Alterations in SAM levels trigger the activation of mitogen-activated protein kinase p38 (MAPK14) and the phosphorylation of MAPK-activated protein kinase 2 (MK2), thereby triggering G1 phase cell cycle checkpoints. In addition, p53 and p27 assist in maintaining cell-cycle arrest, but there is no evidence demonstrating unique connections between the maintenance of cell-cycle arrest and it [43].

### 3.1. Methionine Metabolism and Epigenetic Modification

As more and more studies have been reported, epigenetic modifications become dysregulated during the development of cancer, significantly impacting various biological activities [44]. Methylation is a ubiquitous epigenetic modification found in most cancer cells, involving the covalent attachment of methyl groups [45]. DNA, RNA, and histone methylation are highly conserved in normal cells because of their critical roles in regulating gene expression, genome stability, and the cell cycle [46]. Considering the established fact that SAM is the principal methyl donor in cells, maintaining a stable SAM concentration is essential for preserving the typical methylation patterns of nucleic acids and proteins [47,48,49]. Han et al. [50] further demonstrated that increased SAM levels due to elevated methionine flux lead to genome-wide hypermethylation, which promotes proliferation and inhibits pyroptosis of cancer cells. As a result of the reduced availability of methionine or SAM, the transmethylation rate decreases in cancer cells, resulting in an unstable methylation state, which affects DNA, RNA, and histone methylation modification [41,51].

#### 3.1.1. DNA Methylation

Extensive studies have established that DNA methylation, a prevalent epigenetic modification, plays a vital role in organisms by regulating gene expression, DNA replication, and DNA repair [52,53]. Abnormal DNA methylation patterns are distinctive features of cancer cells and significant contributors to carcinogenesis, involving genome-wide DNA hypomethylation and hypermethylation of specific cancer-related genes [34,54,55]. Genome-wide hypomethylation is associated with increased chromosomal instability, rendering it susceptible to DNA damage and aneuploidy, resulting in heightened cancer heterogeneity and evolution [56]. Conversely, hypermethylation modifications mainly occur at CpG islands within gene expression regulatory elements, suppressing relevant gene expression, impairing DNA damage repair, ultimately promoting tumorigenesis and cancer cell proliferation, and impacting the cancer cell cycle and apoptosis [52,57,58,59].

DNA methylation processes are strongly associated with methionine metabolism in cancer cells [60]. In head and neck squamous cell carcinoma (HNSCC) cells, a decrease in methyl donors disrupts the methylation balance. Methyl donor deficiency leads to elevated expression of DNA methyltransferase 3A (DNMT3A) and Ten-eleven translocation 1 (TET-1), both of which contribute to DNA methylation modification of HNSCC. This results in a decreased methylation of pro-apoptotic factors, such as death-associated protein kinase 1 (DAPK1) and p53 upregulated modulator of apoptosis (PUMA), along with an increase in expression, ultimately resulting in reduced proliferation capacity, impaired migration ability, and increased apoptosis [61]. Nevertheless, this effect can be reversed by supplementing with exogenous methionine. Additionally, studies conducted both in vitro and in vivo have demonstrated that methionine deficiency reduces the expression of long non-coding RNA (lncRNA) PVT1 and influences its interaction with DNMT1, leading to the demethylation of the promoter of BCL2 interacting protein 3 (BNIP3), thereby inhibiting the proliferation of gastric cancer cells [62].

Methionine or SAM not only influences nuclear DNA methylation but also impacts the methylation of mitochondrial DNA (mtDNA). For example, in cervical cancer cells, overexpression of the transporter SLC25A26, responsible for transporting SAM across the mitochondrial membrane, resulted in increased SAM levels in mitochondria and decreased SAM availability in the cytosol. Hypermethylation of mitochondrial DNA decreases respiratory complex expression, diminishes mitochondrial ATP production, and increases cytochrome c synthesis. Therefore, altered SAM availability in the cytosol and mitochondria contributes to the apoptosis of cervical cancer cells [63].

#### 3.1.2. RNA Methylation

Approximately 170 RNA epigenetic modifications have been identified, with RNA methylation modifications constituting two-thirds of the total [64,65]. RNA methylation, a widespread post-transcriptional modification in coding and non-coding RNA, is crucial in regulating diverse biological processes, including but not limited to RNA transcription, splicing, structural conformation, and stability [64,65,66]. Aberrant RNA methylation is intricately associated with the occurrence of cancer, exerting a substantial influence on cancer progression, metastasis, and displaying a robust correlation with patient prognosis [67,68].

Current investigations primarily focus on unraveling the effect of methionine or SAM on m^6^A RNA modification. Beyond regulating MAT2A expression and activity to stimulate SAM synthesis, mTORC1 facilitates the translation of Wilms’ tumor 1-associated protein (WTAP) mRNA, resulting in upregulation of WTAP protein [18]. Consequently, limiting exogenous methionine can decelerate cancer progression by concomitantly inhibiting SAM synthesis and m^6^A RNA modification. Another recent advance has uncovered that methionine modulates the expression of immune checkpoint molecules. Specifically, methionine facilitates m^6^A RNA modification and the translation of PD-L1 and V-domain immunoglobulin (Ig) suppressor of T cell activation (VISTA) in cancer cells, thereby suppressing anti-cancer immunity [69]. Li et al. [70] discovered that methionine, synthesized by pericytes in TME, participates in the stabilization of m^6^A modifications in the mRNA of ATPase family AAA domain-containing protein 2 (ATAD2). ATAD2 facilitates the development of clear cell renal cell carcinoma (ccRCC) and resistance to tyrosine kinase inhibitors (TKIs) by collaborating with SSY-Box transcription factor 9 (SOX9) to form super-enhancers in ccRCC cancer stem cells (CSCs). Zhou et al. [71] demonstrated that a positive feedback loop between m^6^A RNA modification reader IGF2BP2 and the methionine transporter SLC7A5 promotes radiation resistance in lung cancer through the AKT/mTOR pathway. ESCC cells can uptake large amounts of methionine to synthesize SAM, which in turn stabilizes the mRNA and enhances the protein expression of the oncogene NR4A2 via the “METTL3-RNA m^6^A-IGF2BP2” axis, ultimately facilitating ESCC progression [27]. Despite this, less attention has been given to other RNA methylation modifications, such as 5-methylcytosine (m^5^C) and 7-methylguanosine (m^7^G), necessitating a more comprehensive study.

#### 3.1.3. Histone Methylation

Histone methylation refers to the process of methylation that occurs on the N-terminal lysine, arginine, or histidine residues of H3 and H4 histones [72]. This epigenetic process is dynamically reversible and regulated by numerous enzymes [73,74,75]. Histone methylation can alter how histones interact with chromatin and regulate chromatin structure and function, preserving genome integrity and controlling gene expression by influencing transcriptional levels within promoter regions [76,77,78].

It is requisite to highlight that anomalous histone methylation or demethylation of cancer-related genes profoundly influences tumorigenesis and development [74]. Several studies have verified the intricate association between histone methylation patterns, tumor malignancy, and methionine dependence [2,38,79,80,81]. During the transition of methionine-dependent osteosarcoma cells to a methionine-independent state, an increase in trimethylation of histone H3 lysine 9 (H3K9me3) and histone H3 lysine 27 (H3K27me3) is observed, alongside a decrease in methylation at sites such as H3K4me3, H3K36me3, and H3K79me3 [2]. Indeed, changes in histone methylation vary across different cancer cell types under methionine or SAM restriction. Correspondingly, deficiencies in methionine or SAM reduce histone methylation, including H3K4me3, H3K9me2, H3K27me3, and H3K36me3, and can significantly reverse drug resistance in lung cancer [37,82]. Hence, the inhibition of methionine–SAM metabolism significantly reduces histone methylation levels in cancer cells, culminating in pronounced anti-cancer effects [33,36,83,84].

Further studies support the concept that methionine perturbs the epigenome by influencing histone methylation. Reduced methionine and SAM levels induce heterochromatin reduction through interference with DNA methylation. In addition, it results in increased satellite RNA expression by decreasing histone H3K9 and H4K20 methylation levels. These alterations initiate replication stress related to R-loops and chromosomal abnormalities, ultimately culminating in DNA damage and apoptosis in cancer cells [85,86]. Furthermore, methionine impacts cancer cell susceptibility to ferroptosis by modulating histone methylation levels. Elevated SAM levels upregulated ACSL3 expression through enhanced histone H3K4me3 modification in the promoter region, conferring resistance to ferroptosis in cancer cells [87].

*EMSY*, a vital biomarker gene of CSCs in triple-negative breast cancer (TNBC), induces the BRCAness phenotype. EMSY binds competitively to the Jumonji C (JmjC) domain of KDM5B, reshaping methionine metabolism and enhancing CSC characteristics by promoting the methylation modification of H3K4. This interaction suggests that PARP inhibitors combined with methionine restriction may synergize against TNBC stem cells [88]. Additionally, methionine deficiency combined with HDAC2 inhibition, which reduces H3K27me3 levels and increases H3K27ac levels at E-cadherin promoters, inhibits gastric cancer cell migration, invasion, and metastasis [89].

Methionine deficiency can adversely affect the immune function of immune cells by reducing the histone methylation modifications. Methionine restriction leads to the loss of histone H3K79 dimethylation (H3K79me2), impeding STAT5 expression and thereby compromising the functionality of CD8^+^ T cells [90]. Additionally, deleted H3K79me2 upregulates PD-1 expression through inhibiting adenosine 5′-monophosphate (AMP)-activated protein kinase (AMPK) suppression, ultimately attenuating CD4^+^ T cell immune responses [91]. Moreover, methionine insufficiency causes promoter-specific H3K4 trimethylation loss, which disrupts CD4^+^ helper T (Th) cell function [92].

#### 3.1.4. Other Methionine-Related Modifications

Post-translational modifications (PTMs) expand the functional diversity of the proteome through the covalent addition of functional groups or proteins, proteolytic cleavage of regulatory subunits, or degradation of entire proteins [93]. Methionine, as a primary methyl donor, is involved in protein methylation. Methionine promotes the asymmetric dimethylation of YAP R124 (YAP R124me2a), catalyzed by PRMT1, stimulates the transcriptional activation of SLC43A2, and establishes a positive feedback loop to enhance methionine uptake by cancer cells [94]. According to the prevailing view, elevated methionine levels are directly associated with the accumulation of homocysteine, which can modify protein through lysine N-homocysteinylation [95]. To date, studies focusing on colorectal cancer (CRC) have demonstrated that organ-specific colonic lysine homocysteinylation potentially promotes the onset of CRC by hindering DNA damage repair [96]. Additionally, the decomposition product of cysteine, α-ketobutyric acid, can be further transformed into succinyl-CoA to regulate the modification of succinylation [97]. Succinylation is a PTM that modulates cancer progression by affecting various substrate targets and signaling pathways [98,99,100]. This modification is closely linked to metabolic reprogramming, altering the structure or activity of metabolism-related proteins [101]. As mentioned above, there is excellent potential for exploring the relationship between methionine and succinylation modification, which needs further studies.

### 3.2. Methionine Metabolism and Ferroptosis

Ferroptosis, a novel form of iron-dependent cell death, is distinct from apoptosis, cell necrosis, and autophagy [102]. Cysteine is transported to the cell via the system xc (xCT, SLC3A2, and SLC7A11) in the cell membrane, which produces glutathione (GSH) to mitigate lipid peroxide by glutathione peroxidase 4 (GPX4) [103,104]. Consequently, the inhibitors of the system xc and the absence of extracellular cystine can result in GSH deficiency, a fundamental characteristic of ferroptosis, and trigger a cascade of events, including producing reactive oxygen species (ROS) and ferroptosis [102,105,106]. Precise regulation of ROS levels is critical for cell survival. Elevated ROS levels in cancer cells disrupt the redox balance, leading to lipid peroxidation and oxidative stress, resulting in genomic instability, mutations, tumorigenesis, and metastasis [107,108]. Methionine restriction has been shown to exert several effects, including lowering levels of antioxidant NADPH, elevating ROS levels, and decreasing reduced/oxidized glutathione ratios [109,110]. These changes collectively inhibit cancer cell growth and enhance their susceptibility to multiple drugs [109,110,111].

An increasing number of researchers are focusing on investigating the interaction between methionine restriction and ferroptosis. Methionine restriction has the potential to decrease intracellular cysteine levels and promote ferroptosis. It is observed that methionine/cystine restriction (MCR) inhibits the progression of ESCC by inducing ferroptosis both in vivo and in vitro. MCR triggers ferroptosis via a positive feedback loop involving the methionine transporter SLC43A2 and NF-κB signaling pathways. Decreased methionine uptake or lowered SLC43A2 expression results in the inactivation of the NF-κB signaling pathway, which, in turn, leads to the downregulation of SLC43A2 and GPX4, thereby further reducing methionine uptake and facilitating ferroptosis [23]. Further supporting the idea is evidenced by the combination of intermittent methionine deprivation, systemic Xc inhibitors, and programmed cell death protein 1 (PD-1) blockade, demonstrating significant anti-cancer effects [112]. Mechanistically, intermittent methionine restriction can expedite ferroptosis in cancer cells by inducing the transcription and expression of cation transport regulator homolog 1 (CHAC1), which is a γ-glutamylcyclotransferase that specifically targets GSH into 5-OH and Cys-Gly [112,113]. Interestingly, another finding in the study is that prolonged methionine restriction reverses the depletion of GSH by inhibiting the expression of CHAC1 in cancer cells [112]. Supporting this view is that prolonged methionine restriction could counteract the enhancement of ferroptosis induced by System XC inhibitors or cystine deprivation [114,115]. Additionally, SAM contributes to methylation-dependent ubiquinone synthesis, facilitating lipid peroxide accumulation and ferroptosis [116]. As a consequence, exploring the relationship between methionine and ferroptosis is far from abundant, and further studies need to be conducted.

### 3.3. Methionine Metabolism and Synthetic Lethality

**Figure 2:**
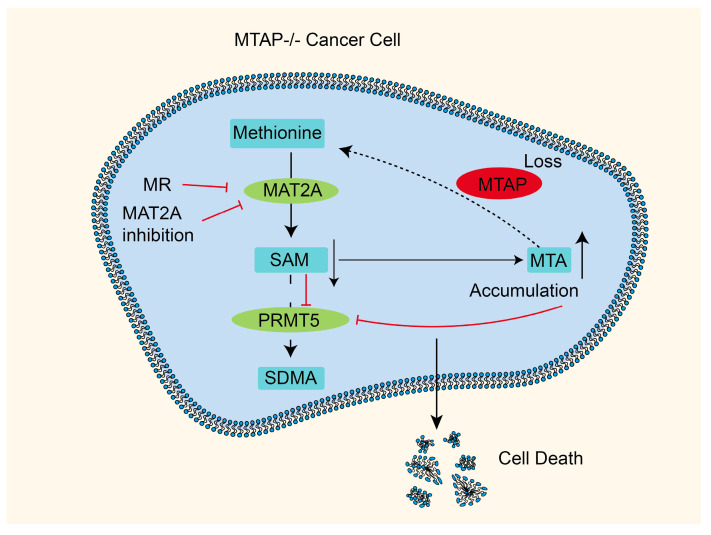
The relationship between methionine restriction and synthetic lethality. The inefficacy of utilizing MTA leads to its substantial accumulation in MTAP-deficient cancer cells. Subsequently, inhibiting MAT2A can diminish SAM production, effectively impeding the activity of PRMT5 and thereby prompting tumor cell death. Vertical black arrows indicate participation in promoting this process, while red arrows indicate inhibition.

An overwhelming body of evidence suggests that MTAP and the cancer suppressor gene cyclin-dependent kinase inhibitor 2A (CDKN2A) are closely situated and concurrently absent in a subset of cancers [117,118]. Recent studies have identified that the absence of MTAP in cancer cells renders them more susceptible to PRMT5 and MAT2A depletion [119,120,121]. This phenomenon can be attributed to a complex relationship within methionine metabolite regulation. Firstly, MTA, the downstream metabolite of SAM, accumulates excessively due to the deletion of MTAP, inhibiting the function and activity of protein arginine methyltransferase 5 (PRMT5) [117]. Secondly, the decreased SAM levels because of restricted MAT2A further impair PRMT5 activity [122]. The high MTA and low SAM environments diminish PRMT5 function and activity in MTAP-deficient cancers. Remarkably, PRMT5 is implicated in various cellular functions involving cell growth, proliferation, and migration, such as its involvement in non-homologous end joining (NHEJ) repair through regulating 53BP1 protein levels via methylation [123,124,125,126,127,128,129,130]. Also, PRMT5 exhibits oncogene-like properties by downregulating the expression of cancer suppressor genes and promoting oncogene expression [131]. For example, it can promote pancreatic cancer progression by inhibiting the cancer suppressor protein 7FBW7 expression, which contains F-box/WD repeats [132]. MAT2A inhibitors efficiently suppress the activity of MAT2A, leading to a substantial decline in SAM levels and exhibiting robust anti-proliferative effects through synthetic lethality in MTAP-deficient cancer cells [117,121,133]. Additionally, the use of recombinant r-METase or implementing a direct methionine restriction diet can yield similar effects **(**Figure 2) [84,133].

## 4. Methionine and Tumor Microenvironment

Cancer constitutes a complex ecosystem comprising cancer cells and the tumor microenvironment (TME), which includes components such as the extracellular matrix, immune cells, and cytokines [134]. Preclinical studies indicate that methionine enhances antitumor immunity through microbiota-mediated immune cell activation [135]. Emerging evidence demonstrates that methionine metabolism plays a pivotal role in modulating antitumor immunity through its profound influence on TME. Malignant cells engage in metabolic competition with immune cells for methionine uptake, creating an immunosuppressive TME. Additionally, methionine and its downstream metabolites critically regulate immune cell gene expression, redox homeostasis, and cell proliferation, ultimately influencing the ability of immune cells to mount an effective anti-cancer immune response [90,92,136].

Mounting evidence illustrates the competition between cancer cells and T cells for methionine through the amino acid transporter on cell membranes, which leads to the creation of an immune-suppressive microenvironment and compromises T cell function and activity [136,137]. The studies indicate that T cells enhance methionine transport from plasma to the cytoplasm by upregulating the methionine transporter SLC7A5. Nevertheless, the transporter SLC43A2, regulated directly by P53 and expressed in cancer cells, exhibits a higher methionine transport capacity than SLC7A5, leading to the preferential uptake of cancer cells [85,137,138]. Collectively, these dynamic results resulted in decreased methionine availability in immune cells, impairing the function of CD8+ T cells, CD4+ T cells, and CD4+ helper T (Th) cells through dysregulation of histone methylation patterns [36,90,91,92]. Subsequent findings have identified additional potential regulatory mechanisms. Metabolic reprogramming in cancer cells upregulates the expression of methionine metabolism-related genes, such as *AHCY*, which causes an accumulation of SAM/MTA within the TME, consequently influencing chromatin reprogramming and inducing dysfunction in T cells [139].

**Figure 3:**
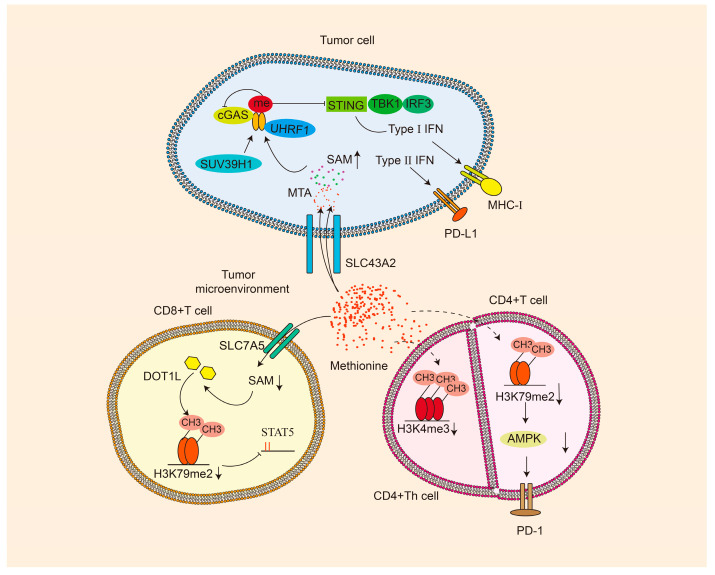
The relationship between methionine and tumor microenvironment. Methionine exerts an inhibitory effect on the anti-tumor immune function of immune cells by influencing the dynamics of the tumor microenvironment. Decreased SAM levels compromise the immune function of CD8+ T cells, upregulate PD-1 expression in CD4+ T cells, and impair the proliferation and cytokine production of CD4+ Th cells.

Methionine participates in the methylation of cyclic GMP-AMP synthase (cGAS) by methyltransferase SUV39H1. The interaction of methylated cGAS with ubiquitin-like with plant homeodomain and RING finger domains 1 (UHRF1) promotes chromatin isolation of cGAS and inhibits its function [140]. Methionine restriction induces MHC-I expression on cancer cell surfaces by activating the cGAS-stimulator of interferon genes (STING)- type I interferon (IFN-I) signaling pathway [140,141]. It has also been shown that methionine restriction can induce the *PD-L1* gene expression on cancer cell surfaces by activating the type II interferon signaling pathway [141]. Simultaneously, the in-depth in vivo study has shown that combining dietary methionine restriction with immune checkpoint inhibitors targeting CTLA-4 and PD-1 is significantly more effective than monotherapy [141]. Additionally, it has been confirmed that under methionine-deficient culture conditions, the suppression of immune function correlates with heightened DNA damage and chromosomal aberrations in mononuclear/macrophage cells (Figure 3) [142].

Given the intricate composition of the TME, a comprehensive evaluation of the effects of methionine restriction on cancer cells and immune cells is essential. Exorbitant methionine restriction may impair the function of tumor-infiltrating CD8+ T cells and influence cancer progression by affecting mechanisms such as hydrogen sulfide (H_2_S) related immune signaling [143,144]. Recent studies have indicated that methionine restriction inhibits CAR-T cell killing capacity, reduces cytokine release, diminishes central memory T phenotype, and upregulates exhaustion markers. Nevertheless, the reduction in SAM level results in a downregulation of m^5^C modification and a decrease in NKG7 expression, further reducing the toxicity of CAR-T cells [145]. In addition, microbial dysbiosis in the TME contributes to maintaining the cancer phenotype by directly supplying methionine to cancer cells. Research findings about lung adenocarcinoma (LUAD) show that bacteria are abundant at methionine synthesis pathways while displaying diminished SAM metabolic pathways in the TME [146].

## 5. Therapeutic Strategies Targeting Methionine in Cancer

Targeted cancer metabolism has emerged as a novel approach to cancer treatment, owing to its significant influence on treatment responses and potential to mitigate treatment-related side effects. The metabolic reprogramming of cancer cells is characterized not only by excessive dependence on methionine but also by an increased demand for other amino acids such as glutamine and serine, the phenomenon referred to as glutamine or serine dependency [147,148,149]. As an essential carbon and nitrogen donor, glutamine fuels the tricarboxylic acid (TCA) cycle via α-ketoglutarate (α-KG) generation while simultaneously supporting GSH biosynthesis [150,151]. Serine provides key one-carbon units for biosynthetic pathways, supporting cancer cell growth by generating glutathione, nucleotides, and phospholipids [148,149]. Cancer cells exhibit extraordinary metabolic plasticity, adapting to nutrient stress through multifaceted gene regulatory networks. Such adaptations maintain viability and proliferative capacity despite restrictions in glutamine or serine metabolism [152]. In contrast to glutamine or serine dependency, methionine dependency constitutes an obligate metabolic vulnerability in cancer cells due to its essentiality and irreplaceability by homocysteine, offering unique opportunities for targeted intervention.

Methionine restriction (MR) emerges as a universally effective treatment [153,154]. MR can be achieved by directly adjusting the diet to reduce methionine uptake or employing enzymes involved in methionine catabolism. Alternatively, it can be indirectly achieved by using inhibitors that target methionine uptake and metabolic pathways. Furthermore, combining MR with specific chemotherapy drugs, immunosuppressants, and other treatments promises a therapeutic strategy [153,155,156,157]. Hoffman et al. [158,159] discovered that MR can inhibit the cancer cell cycle, leading to S/G2 phase arrest and enabling chemotherapy drugs such as cisplatin to exert their effects during this period. This combined approach to MR and chemotherapy for cancer treatment is referred to as the Hoffman protocol. In the following sections, we briefly summarize MR therapies, combination therapies, and their inherent limitations. The optimal therapeutic strategy may vary depending on the cancer type, patient characteristics, and potential molecular alterations. However, cancer cells are highly adaptable and may also develop resistance to methionine-targeting therapies. To compensate for methionine deprivation, cancer cells can upregulate alternative metabolic pathways or increase the expression of methionine transporters. Therefore, the concurrent implementation of multiple methionine restriction strategies may enhance clinical outcomes (Table 1).

**Table 1:** Methionine restriction treatment strategy.

Treatment	Compounds	Clinical Stage	Applications	Reference
Direct methionine restriction	MRD/DMR	phase II Clinical	MRD combined with cystemustine treatment for melanoma or glioma	[160]
	rMETases	phase I Clinical	High-stage cancer patients	[161]
	hCGL	Preclinical	Prostate cancer	[162]
	MGL S3	Preclinical	Neuroblastoma, breast cancer, non-small cell lung carcinoma, colon cancer and epidermoid carcinoma	[163,164]
	Erymet	Preclinical	Colorectal carcinoma, glioblastoma, gastric and pancreatic cancers	[83,165]
	SYNB1353	Preclinical	/	[166]
	SGN1	phase I/IIa Clinical	Advanced solid tumor patients	[167]
Indirect methionine restriction	Cycloleucine	Preclinical	/	[168,169]
	PF-9366 (MAT2A inhibitor)	Preclinical	Lung carcinoma, leukemia	[15,170]
	FIDAS-3/FIDAS-5 (MAT2A inhibitor)	Preclinical	Colon cancer, multiple myeloma	[171,172]
	AGI-24512/AGI-25696/AG-270/AGI-41998 (MAT2A inhibitor)	Preclinical/phase I Clinical	Advanced solid tumors or lymphoma with MTAP loss	[117,121,173,174]
	IDE397 (MAT2A inhibitor)	phase I/II Clinical	Solid tumors harboring MTAP deletion	[175,176]
	BCH (SLC7A5 and SLC43A2 inhibitor)	Preclinical	Lung cancer, gastrointestinal cancer	[94,177]
	KMH-233 (SLC7A5 inhibitor)	Preclinical	Breast cancer cells	[178]
	JPH203 (SLC7A5 inhibitor)	Preclinical/phase I Clinical	Prostate and thyroid cancer	[179,180,181]

### 5.1. Direct Methionine Restriction

#### 5.1.1. Methionine Restriction Diet

The dietary limit of methionine intake is often termed a methionine restriction diet (MRD), or methionine-restricted diet, or dietary methionine restriction (DMR) therapy. Animal experiments have revealed that MRD therapy significantly inhibits cancer cell proliferation, induces apoptosis, and reduces cancer volume in vivo compared to the control group [182,183,184]. Moreover, the combination of MRD with chemotherapy drugs and immune checkpoint inhibitors has notably surpassed the impact of single-agent therapy [141,185]. It is worth noting that MRD should not fall below a minimum concentration of 0.12% since levels lower than this threshold could trigger a distinctive response to essential amino acid deficiency, involving anorexia and rapid weight loss [186]. A phase II clinical trial conducted on patients with melanoma or glioma demonstrated promising clinical and translational prospects for MRD. Specifically, 22 patients received MRD in addition to cystemustine (60 mg/m^2^) every two weeks, with a median treatment duration of 8 weeks. The results indicated that patients did not experience any significant adverse events during treatment. The median disease-free survival (DFS) is 1.8 months, the median overall survival (OS) is 4.6 months, and plasma methionine concentration decreased by 40% [160].

#### 5.1.2. Methionine γ Lyase

Methionine γ lyase (MGL, EC 4.4.1.11), commonly known as methioninase, is first isolated from bacteria such as *Clostridium sporogenes*, demonstrating potential anti-cancer effects [187,188]. MGL is widely distributed across diverse microorganisms and demonstrates significant similarity in biochemical characteristics [189,190,191]. The mechanism of MGL relies on pyridoxal 5′-phosphate (PLP), catalyzing the direct conversion of L-methionine into methane mercaptan, α-ketobutyrate, and ammonia. Moreover, MGL exhibits diverse catalytic functions, encompassing α and γ-elimination as well as γ-substitution reactions with methionine and its analogs [192,193,194].

Researchers have been diligently endeavoring to advance the clinical application of methioninase, with one notable success linked to recombinant methioninase (rMETase), manifesting heightened catalytic methionine activity in vivo and in vitro experiments. Moreover, it exhibits minimal toxic side effects, evokes no immune response, and is conducive to large-scale production [195,196,197]. Both cell lines and patient-derived orthotopic xenograft (PDOX) models have elucidated the effectiveness of rMETase against cancers [159,198,199,200,201,202,203]. A pilot phase I clinical trial suggests that intravenous (IV) therapy of rMETase has proven safe for high-stage cancer patients and effective in reducing methionine levels in patient serum [161]. Substantial evidence indicates that the anti-cancer effect of oral rMETase(o-rMETase) may exceed that of intravenous infusion of rMETase [204,205]. Oral rMETase, either alone or in combination with other drugs, exerts a remarkable effect in inhibiting the progression of various cancers, including pancreatic, gastrointestinal, prostate, and ovarian [202,206,207,208,209,210,211]. Unfortunately, clinical trials relevant to rMETase have encountered delays, remaining in the phase I clinical stage. The utilization of rMETase in cancer patients is relatively underreported, necessitating future investigations involving a broader cohort of advanced cancer patients to assess the effectiveness.

Furthermore, other biologics are currently under development for MR. The human cystathionine-γ-lyase (hCGL) has been engineered to have methionine degradation capabilities, exhibits low immunogenicity, and significantly inhibits the growth of prostate cancer [162,212]. Notably, alternative endeavors like MGL S3, a genetically engineered protein formed by combining MGL with an epidermal growth factor (EGF)-like peptide, bind directly to cancer cells, inhibiting their proliferation and enhancing their sensitivity to EGFR inhibitors [163,164]. In addition, Erymet, where MGL is encapsulated in red blood cells, not only mitigates the immune response but also significantly improves the pharmacokinetics and effectiveness of MGL [83,165]. Unfortunately, no clinical efficacy data for hCGL, MGL S3, and Erymet have been reported. Recently, Perreault et al. found that the live probiotic SYNB1353, a derivative of Escherichia coli Nissle 1917, reduced plasma methionine levels by 26% in healthy volunteers and demonstrated high safety and tolerability [166].

SGN1 (SalMet-Vec) is equipped with L-methioninase derived from an attenuated salmonella vector, which exerts an anti-tumor effect by depriving tumor cells of methionine. RNA-seq analyses have revealed that SGN1 downregulates the expression of various genes associated with cell growth, migration, and invasion. Currently, the clinical trials of SGN1 in phase I/IIa for solid tumors are in order (NCT05038150 and NCT05103345). As shown in animal studies, both intratumoral and intravenous administration of SGN1 can inhibit tumor growth by activating CD8+ T cell responses in TME [167,213].

### 5.2. Indirect Methionine Restriction

#### 5.2.1. MAT2A Inhibitors

Targeting MAT2A has been proven to be an effective strategy in inhibiting SAM biosynthesis, thereby indirectly achieving MR and influencing subsequent biological functions associated with methionine and SAM [121]. Recently, numerous MAT2A inhibitors have been reported, including cycloleucine, FIDAS-3, FIDAS-5 (Abmole, Houston, TX, USA), PF-9366 (Pfizer compounds, New York, NY, USA), AGI-24512, AGI-25696, AG-270, AGI-41998 (Agios compounds, Cambridge, MA, USA), IDE397 (Ideaya Biosciences, South San Francisco, CA, USA), S-095035 (Servier, Suresnes, France), and ISM3412 (InSilico Medicine Hong Kong Limited, Hong Kong, China). Among these, AG-270, IDE397, S-095035, and ISM3412 are currently undergoing clinical trials to treat MTAP-deficient solid tumors and lymphomas [15,121,171,175,176]. Prior studies have demonstrated that using MAT2A inhibitors alone can induce MR, reduce SAM levels, and impede the progression of various cancers, including breast cancer, lung cancer, and multiple myeloma [37,112].

##### Cycloleucine

Cycloleucine is a non-metabolizable amino acid, widely utilized as a precursor in peptide synthesis. It is a specific inhibitor of S-adenosylmethionine-mediated methylation, acting as a methionine analog to reduce S-adenosylmethionine synthesis by inactivating methionine adenosyltransferase. Additionally, it acts as an antagonist at the glycine allosteric site, where the N-methyl-D-aspartate (NMDA) receptor is linked to glycine [168,169]. However, cycloleucine exhibits a weak binding affinity for MAT2A and may inhibit other cellular pathways, leading to limited research interest.

##### PF-9366

As the first small-molecule allosteric inhibitor of MAT2A, PF-9366 binds to an allosteric site on MAT2A and alters the MAT2A activity, resulting in increased substrate affinity and reduced enzyme turnover [15]. As a nonspecific inhibitor, PF-9366 competes with MAT2B to bind MAT2A, effectively inhibiting the production of SAM in cells. PF-9366 induces reduced SAM levels and reduced methylation of the MAT2A promoter, driving adaptive upregulation of *MAT2A* mRNA and protein expression to compensate for the functional inhibition of MAT2A. Compensatory regulation of MAT2A expression resulted in the inability of PF-9366 to effectively inhibit cell proliferation, so relevant studies are limited to preclinical trials [15].

##### FIDAS-3, FIDAS-5

FIDAS-3 and FIDAS-5 are a series of novel stilbene analogs that bind MAT2A specifically by occupying SAM binding sites [171,214]. In vivo experiments have confirmed that both compounds have strong effects and reasonable half-lives with lower toxicity. FIDAS-5 showed better MAT2A inhibitory activity than FIDAS-3 [214]. In addition, FIDAS-5 reduces the proliferation and survival of multiple myeloma (MM) cells in vivo and in vitro by inhibiting mTOR-mediated protein synthesis and can enhance the efficacy of bortezomib, a commonly used chemotherapy drug, in MM therapy [172].

##### Other Small Molecule Compounds

Analysis of a small-molecule compound library, based on the structure of the MAT2A protein, revealed that compound AGI-24512 can effectively inhibit MAT2A activity [121]. Similar to PF-9366, AGI-24512 leads to SAM depletion while MAT2A protein expression is upregulated, because increased METTL16 occupancy on the MAT2A 3′ UTR is sufficient to promote efficient splicing, thereby inducing MAT2A expression through enhanced intron-retaining splicing. AGI-24512 compensates by increasing MAT2A expression, resulting in selective anti-proliferative activity and a dose-dependent decrease in SAM levels in MTAP-deficient cells, whereas its anti-proliferative effects are limited in most MTAP wild-type cells [117,121]. Pharmacokinetic studies revealed that AGI-24512 has poor oral absorption and a short half-life in vivo. AGI-25696, which differs from AGI-24512 by a single functional group modification, exhibits greater stability. It undergoes tautomerism, is weakly acidic, and demonstrates strong plasma protein binding, but still requires a sufficiently high dose for adequate systemic exposure [121].

To further improve the physicochemical properties and cellular activity of the inhibitor, researchers optimized internal hydrogen bonding interactions and designed AG-270. AG-270 has demonstrated strong efficacy in vitro studies, effectively reducing intracellular SAM levels, exhibiting selective anti-proliferative activity in MTAP-null cells, and sensitizing their chemosensitivity to taxanes [121]. Additionally, AG-270 induces DNA damage and impairs DNA repair activity in MTAP-deficient cells, suggesting that MAT2A inhibition could enhance the efficacy of cancer therapies targeting DNA damage responses [117]. A previous phase I clinical trial for patients with advanced solid tumors or lymphomas harboring MTAP deficiency showed that AG-270 effectively reduced SAM concentrations and SDMA levels in tumor cells, leading to a selective growth disadvantage in homozygous MTAP-deficient cancer cells (NCT03435250). However, the trial is discontinued due to hepatotoxicity at higher doses, potentially attributed to AG-270-induced UGT1A1 inhibition, leading to elevated bilirubin levels, or partial inhibition of MAT1A [173].

Other novel MAT2A inhibitors, such as AGI-41998, exhibit potent inhibitory activity and favorable brain permeability, significantly suppressing cancer growth in xenograft models. However, AGI-41998 also activates the human pregnane X receptor (hPXR), leading to increased cytochrome P450 (CYP450) expression and undesirable drug interactions, which have hindered its further development [174].

IDE397, another effective oral MAT2A inhibitor, has been approved by the FDA to enter two clinical trials [175]. The first is a phase I study (NCT04794699), designed to evaluate the safety, pharmacokinetics, and pharmacodynamics of IDE397 in adult participants with advanced solid tumors. A phase I/II study is evaluating the safety, tolerability, pharmacokinetics, pharmacodynamics, and efficacy of IDE397 in combination with the PRMT5 inhibitor AMG 193 in patients with advanced solid tumors (NCT05975073).

#### 5.2.2. SLC Transporters Inhibitors

Methionine is transported into the cell via SLC transporters to exert biological functions; antagonizing these transporters may contribute to MR. 2-Amino-2-norbornanecarboxylic acid (BCH), as an L-type transporter inhibitor, suppresses SLC43A2 activity, thereby restricting methionine uptake and inhibiting tumor cell growth [94]. Additionally, BCH acts as a low-affinity inhibitor of SLC7A5 by selectively binding to the inwardly open conformation of LAT1 [177]. KMH-233 is a selective, slowly reversible LAT1 inhibitor that has been shown to enhance the anti-proliferative effects of cisplatin and bestatin in breast cancer cells [178]. JPH203 (Nanvuranlat/KYT-0353) is a tyrosine analog based on the structure of the thyroid hormone triiodothyronine (T3), a substrate for the LAT1 and LAT2 transporters. As a high-affinity LAT1 inhibitor, JPH203 effectively suppresses the proliferation of prostate and thyroid cancer cell lines in vitro [179,181]. It has entered a phase I clinical trial (UMIN000016546) and has demonstrated good tolerability at lower doses [180].

### 5.3. Adaptive Resistance of Methionine Restriction Therapy

MR has effectively reduced chemotherapy resistance across multiple cancer models [37,158,199,202]. Although several components linking methionine metabolism to proliferative control have been identified, the underlying mechanisms remain incompletely characterized. The potential development of acquired resistance to sustained MR requires thorough investigation. The metabolic complexity of tumors suggests that MR may induce cellular stress responses, potentially driving metabolic adaptation through the activation of compensatory pathways. For example, tumor-specific genetic alterations can significantly influence cancer cell sensitivity to methionine restriction therapy [215].

As previously discussed, the conversion of serine to glycine during the folate cycle facilitates the remethylation of homocysteine, thereby regenerating partial methionine. Concurrently, ATP derived from serine catabolism contributes to the maintenance of the methionine cycle in cancer cells. These findings suggest that cancer cells have the ability to synthesize methionine endogenously, even under conditions of methionine deficiency. Moreover, when amino acid availability is limited, cancer cells frequently employ macropinocytosis and lysosomal proteolysis to acquire proteinogenic amino acids from extracellular proteins [216]. Tumor-associated macrophages may also acquire methionine through the phagocytosis of apoptotic cells, followed by exocytic release, which supports cancer progression and metastatic dissemination [217]. Although no tumor cells have demonstrated resistance to MR therapy, these adaptive pathways may confer latent resistance to prolonged MR treatment.

Indirect methionine restriction strategies, particularly those that inhibit cancer-selective methionine transporters, remain susceptible to resistance mechanisms. The incomplete characterization of SLC transporters’ substrates, combined with their essential function in nutrient and drug uptake, suggests that dysregulation of these transporters can significantly disrupt cellular homeostasis [218]. Furthermore, the remarkable functional redundancy within the SLC superfamily may allow for compensatory methionine uptake when primary transport systems are inhibited.

### 5.4. Clinical Translation of Methionine Restriction Therapy

Except for its established role in inhibiting tumor growth, MR exhibits pleiotropic effects on various physiological systems, including the regulation of aging processes, the maintenance of metabolic homeostasis, and the modulation of insulin signaling pathways [219]. The clinical implementation of DMR as a standalone therapy faces two fundamental challenges. The first challenge is to achieve maximal anti-cancer efficacy while avoiding systemic metabolic dysregulation and adverse physiological consequences. The second involves the complex amino acid composition of food and the difficulty of ensuring adequate protein nutrition while eliminating methionine.

Consequently, methioninase or indirect methionine restriction may offer superior efficacy and tolerability compared to dietary interventions. Among the methioninases currently under investigation, rMETase has exhibited potent methionine-depleting activity in preclinical models and early-phase clinical trials, showcasing both safety and preliminary efficacy. The optimized formulation, o-rMETase, exhibits enhanced clinical applicability and holds significant promise for therapeutic development. Subsequent clinical trials involving larger patient cohorts will be essential to establish its therapeutic utility and facilitate clinical translation.

Furthermore, there is a pressing need for innovative precision cancer treatment strategies that selectively disrupt methionine metabolism in malignant cells while minimizing off-target effects on healthy tissues. Current advancements include cancer-targeted delivery of methioninase, such as MGL S3 and SGN1, achieved through genetic engineering or vector-based systems, an approach with substantial translational potential. These targeted modalities have the potential to overcome the limitations of DMR while preserving potent antineoplastic activity.

Several small molecule inhibitors targeting methionine metabolism have advanced to clinical trials, demonstrating promising therapeutic potential. The MAT2A inhibitor, such as IDE397, selectively induces synthetic lethality in MTAP-deficient malignancies, while SLC7A5 inhibitors, such as JPH203, exert anti-cancer activity by blocking SLC7A5. These agents effectively impose indirect methionine restriction and represent promising candidates for metabolic targeting strategies. However, the therapeutic potential of these inhibitors must be validated through rigorous clinical trials to establish both their efficacy and safety profiles in human populations. Two critical considerations arise for these compound inhibitors: the potential for off-target effects that may compromise treatment specificity and the possible disruption of unidentified signaling pathways that could lead to unanticipated biological consequences.

Regardless of the therapeutic approach, a comprehensive evaluation of the patient’s health status and treatment tolerance remains essential [220]. It is also important to adjust chemotherapy regimens and tailor MR strategies personalized to individual patient characteristics. As different malignancies demonstrate heterogeneous responses to MR, the identification of reliable biomarkers is crucial for determining optimal restriction thresholds and predicting both therapeutic outcomes and prognosis. Ultimately, the clinical application of MR therapies necessitates larger-scale randomized trials to more thoroughly investigate the mechanisms of methionine dependency and validate its therapeutic value.

## 6. Discussion

As an essential amino acid, methionine plays a pivotal role in tumorigenesis through multiple mechanisms, including serving as a precursor for epigenetic modifications. It influences malignant progression through direct effects on cancer cell metabolism and by modulating TME. Methionine restriction therapy has emerged as a promising anti-cancer strategy, demonstrating significant therapeutic efficacy with minimal adverse effects in preclinical studies, thereby warranting serious consideration for clinical translation.

This comprehensive review systematically examines the multifaceted relationship between methionine metabolism and tumor biology, including its critical interactions with the tumor microenvironment and the therapeutic potential of targeted methionine interventions. To advance our understanding of methionine dependence and facilitate the clinical translation of methionine restriction therapy, we propose several key thinking: (1) The downstream metabolite of methionine, SAM, may influence the expression of target genes, such as members of the SLC superfamily, through involvement in epigenetic modifications such as m^6^A and m^5^C RNA modification, thereby establishing a regulatory feedback loop that contributes to tumorigenesis. (2) Methionine dependency displays significant heterogeneity across various cancer types. We propose that this heterogeneity may be influenced by multiple factors, including genetic mutations, cancer stage, and the tumor microenvironmental variations. (3) Current studies have primarily focused on the immunomodulatory effects of methionine metabolism on T cells. We hypothesize that methionine metabolism, as a critical component of the immune microenvironment, may also influence other immune cell populations, including natural killer (NK) cells, myeloid lineage cells, and antigen-presenting cells. This expanded perspective may provide novel insights into the relationship between methionine metabolism and immune status, thereby informing the optimization of methionine restriction and immunotherapeutic strategies. (4) Based on current evidence, we propose several potential factors that may underlie resistance to methionine restriction therapy, including metabolic reprogramming and compensatory activity of SLC transporters. We suggest that methioninase or indirect methionine restriction may offer superior clinical translational potential compared to DMR. This perspective may facilitate the clinical advancement and application of targeting methionine therapies and provide a novel framework for future clinical investigations.

## 7. Conclusions

Metabolic reprogramming contributes to the phenomenon of methionine dependence in cancer, which influences malignant traits such as rapid proliferation and evasion of apoptosis. It is crucial to have a comprehensive understanding of the complex relationship between methionine and cancer. The primary features of methionine dependence lie in the elevated transmethylation levels and altered methylation patterns in cancer cells, fundamentally leading to an increased demand for SAM. Moreover, the close link between methionine and other forms of death, such as ferroptosis, should not be ignored. The dysregulation of methionine in the TME and competition for methionine between cancer and immune cells create an imbalance in methionine utilization by immune cells, impairing their ability to exert normal anti-cancer immune functions.

With the growing recognition of methionine’s pivotal role in tumorigenesis and cancer progression, targeting methionine has become a prominent research focus. Preclinical and clinical research conducted thus far indicates that methionine restriction holds promise as a therapeutic approach. Restricting dietary methionine intake or supplementing with methioninase can elicit anti-cancer effects and significantly enhance the efficacy of chemotherapy. Notably, compared to the noticeable anti-cancer effects, the side effects of methionine restriction and methioninase can be overlooked. In summary, the various approaches to methionine restriction require further research to ascertain the optimal treatment regimen, including the duration of treatment, especially given the limited number of patients involved in clinical trials.

## Data Availability

Not applicable.

## Abbreviations

The following abbreviations are used in this manuscript:

SAM	S-adenosyl-methionine
TME	Tumor microenvironment
MATs	Methionine adenosyltransferases
SAH	S-Adenosylhomocysteine
5-MTHF	5-methyltetrahydrofolate
MTA	Methylthioadenosine
MTAP	Methylthioadenosine phosphorylase
5,10-MTHF	5,10-methylene tetrahydrofolate
mTORC1	Mechanistic target of rapamycin complex 1
DNMTs	DNA methyltransferases
CpG	Cytosine–phosphate–guanine
DAPK1	Death-associated protein kinase 1
PUMA	P53 upregulated modulator of apoptosis
BNIP3	BCL2 interacting protein 3
lncRNA	Long non-coding RNA
PET	Positron emission tomography
WTAP	Wilms tumor 1-associated protein
SLC	Solute carriers
PD-L1	Programmed cell death ligand 1
VISTA	V-domain immunoglobulin suppressor of T cell activation
ATAD2	ATPase family AAA domain-containing protein 2
PRMT	Protein arginine methyltransferase
ACSL3	Acyl-CoA synthetase long chain family member 3
TNBC	Triple-negative breast cancer
PARP	Poly (ADP-ribose) polymerase
CSCs	Tumor stem cells
ROS	Reactive oxygen species
MCR	Methionine/cystine restriction
CHAC1	Cation transport regulator homolog 1
MR	Methionine restriction
MAPK14	Mitogen-activated protein kinase P38
MK2	MAPK-activated protein kinase 2
GSH	Glutathione
NF-κB	Nuclear factor kappa B
PD-1	Programmed cell death protein 1
CAR-T	Chimeric antigen receptor T-cell
cGAS	Cyclic GMP-AMP synthase
UHRF1	Ubiquitin-like with plant homeodomain and RING finger domains 1
STING	Stimulator of interferon genes
MGL	Methionine γ lyase
hCGL	Human enzyme cystathionine-γ-lyase
MRD	Methionine restriction diet
rMETase	Recombinant methioninase
